# Compromised dental cells viability following teeth-whitening exposure

**DOI:** 10.1038/s41598-021-94745-w

**Published:** 2021-07-30

**Authors:** Ola Redha, Morteza Mazinanian, Sabrina Nguyen, Dong Ok Son, Monika Lodyga, Boris Hinz, Marianne Odlyha, Ailbhe McDonald, Laurent Bozec

**Affiliations:** 1 Division of Prosthodontics, UCL Eastman Dental Institute, University College London, 21 University Street, London, WC1E 6DE UK; 2 Division of Biomaterials and Tissue Engineering, UCL Eastman Dental Institute, Royal Free Campus, Rowland Hill Street, London, NW3 2PF UK; 3 Faculty of Dentistry, University of Toronto, 124 Edward Street, Room 459, Toronto, ON M5G 1G6 Canada; 4 Department of Biological Sciences, Birkbeck, University of London, Malet Street, London, WC1E 7HX UK

**Keywords:** Biological models, Dental pulp, Dentine, Enamel

## Abstract

This study aimed to assess the viability of dental cells following time-dependent carbamide peroxide teeth-whitening treatments using an in-vitro dentin perfusion assay model. 30 teeth were exposed to 5% or 16% CP gel (4 h daily) for 2-weeks. The enamel organic content was measured with thermogravimetry. The time-dependent viability of human dental pulp stem cells (HDPSCs) and gingival fibroblast cells (HGFCs) following either indirect exposure to 3 commercially available concentrations of CP gel using an in-vitro dentin perfusion assay *or* direct exposure to 5% H_2_O_2_ were investigated by evaluating change in cell morphology and by hemocytometry. The 5% and 16% CP produced a significantly lower (p < 0.001) enamel protein content (by weight) when compared to the control. The organic content in enamel varied accordingly to the CP treatment: for the 16% and 5% CP treatment groups, a variation of 4.0% and 5.4%, respectively, was observed with no significant difference. The cell viability of HDPSCs decreased exponentially over time for all groups. Within the limitation of this in-vitro study, we conclude that even low concentrations of H_2_O_2_ and CP result in a deleterious change in enamel protein content and compromise the viability of HGFCs and HDPSCs. These effects should be observed in-vivo.

## Introduction

**Figure 1:**
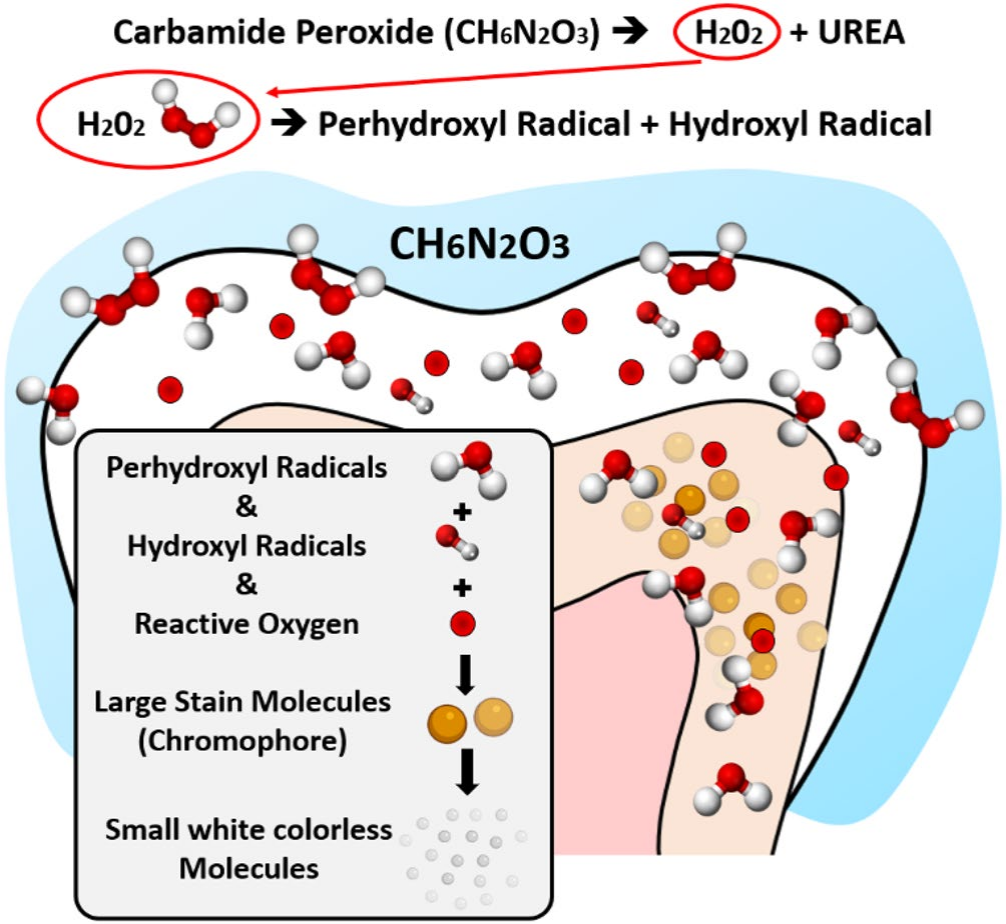
Synopsis of carbamide peroxide teeth-whitening chemistry.

The quest for teeth-whitening reflects patients' demands for superior aesthetics and the considerable advancement in teeth-whitening agents and techniques. Although this procedure is routinely carried out to improve smile aesthetics, there remain two common adverse effects reported in-vivo following vital tooth-whitening therapies: gingival irritation^1^ and postoperative sensitivity^2^. Both these adverse effects are linked directly to the by-products released from the degradation of activated bleaching gels^3,4^. Carbamide Peroxide (CP) is one of the most commonly used treatments for vital home teeth-whitening. CP (CO(NH_2_) H_2_O_2_) is organic, white, and crystalline, and it will break down into hydrogen peroxide (H_2_O_2_) and urea^5,6^. H_2_O_2_ has a low molecular weight and a high oxidative power, favouring its rapid diffusion into enamel prisms and interprismatic spaces^7^. H_2_O_2_ may dissociate into water, reactive oxygen, and free radical species, such as hydroxyl radicals (OH−). In the tooth, the “whitening reaction” is thought to be carried out by H_2_O_2_-derived free radicals breaking down large dentinal chromogenic molecules (chromophores) into smaller molecules with lesser or non-absorbing optical properties, Fig. 1^8,9^. Unfortunately, H_2_O_2_ does not remain confined to the dentin and can reach the pulp chamber mostly by diffusion through dentinal tubules. It has been suggested that upon reaching the pulp, H_2_O_2_ will lead to a decrease of cell proliferation, metabolism, and viability^10^, a reduction of pulp-reparative capacity^11^, tissue necrosis^12^, and finally inducing pulpal pain^13^. An early report suggested that at low concentrations, bleaching agents are not harmful to dental structures^14^. Yet, there is growing in-vitro evidence that at a low concentration (5% and 10%) CP, the deleterious effect of H_2_O_2_-derived free radicals can be detected throughout the dentin and across the pulp chamber^5,15^. Although the use of teeth-whitening agents and techniques are becoming increasingly more popular, there are no studies to date in the scientific literature that have investigated the possible deleterious effect of H_2_O_2_-derived free radicals directly on the dental cells. Performing such experiments directly on patients would jeopardize the vitality of the tooth itself as one would require access to pulpal tissue. Considering the natural function of the dental pulp stem cells in the production of odontoblasts to create reparative dentin and for the pulp itself to support the vitality of the entire tooth, it is critical nonetheless to evaluate how these by-products impact the population of such a critical stem cell reservoir. This in-vitro study aims to evaluate the impact of exposure to a different concentration of commonly used home peroxide-based teeth-whitening on dental cells (HDPSCs and HGFCs) directly or indirectly using a dentin perfusion disc model.

## Materials and methods

### Teeth-whitening agents

Commercial carbamide peroxide (CP) tooth-whitening gels with a concentration of 5%, 10%, 16%, and 35% were obtained directly from the manufacturer (the authors are grateful for the donation of commercial carbamide peroxide (CP) tooth-whitening gels directly from the manufacturer). The CP gel's chemical composition was Water (Aqua), Glycerine, Carbomer (Sodium salt), Hydrogen Peroxide, Sodium Tripolyphosphate, Urea, Potassium Nitrate, Sodium Fluoride, and Aroma. A titrated 5% hydrogen peroxide solution (SIGMA-ALDRICH, USA) was also used as a control for the gingival fibroblast study.

### Collection of tooth samples

A total of 64 intact human premolars and third molars extracted for orthodontic reasons (age group 14–21) were collected for this study under ethical approval (study number 1703) from UCL Eastman BioBank (12/YH/0111). Informed consent was sought from patients and/or parent for the patient under 18 years old for each tooth used in this study. All experimental protocols used in this study were approved by the University College London Eastman BioBank (12/YH/0111).

**Figure 2:**
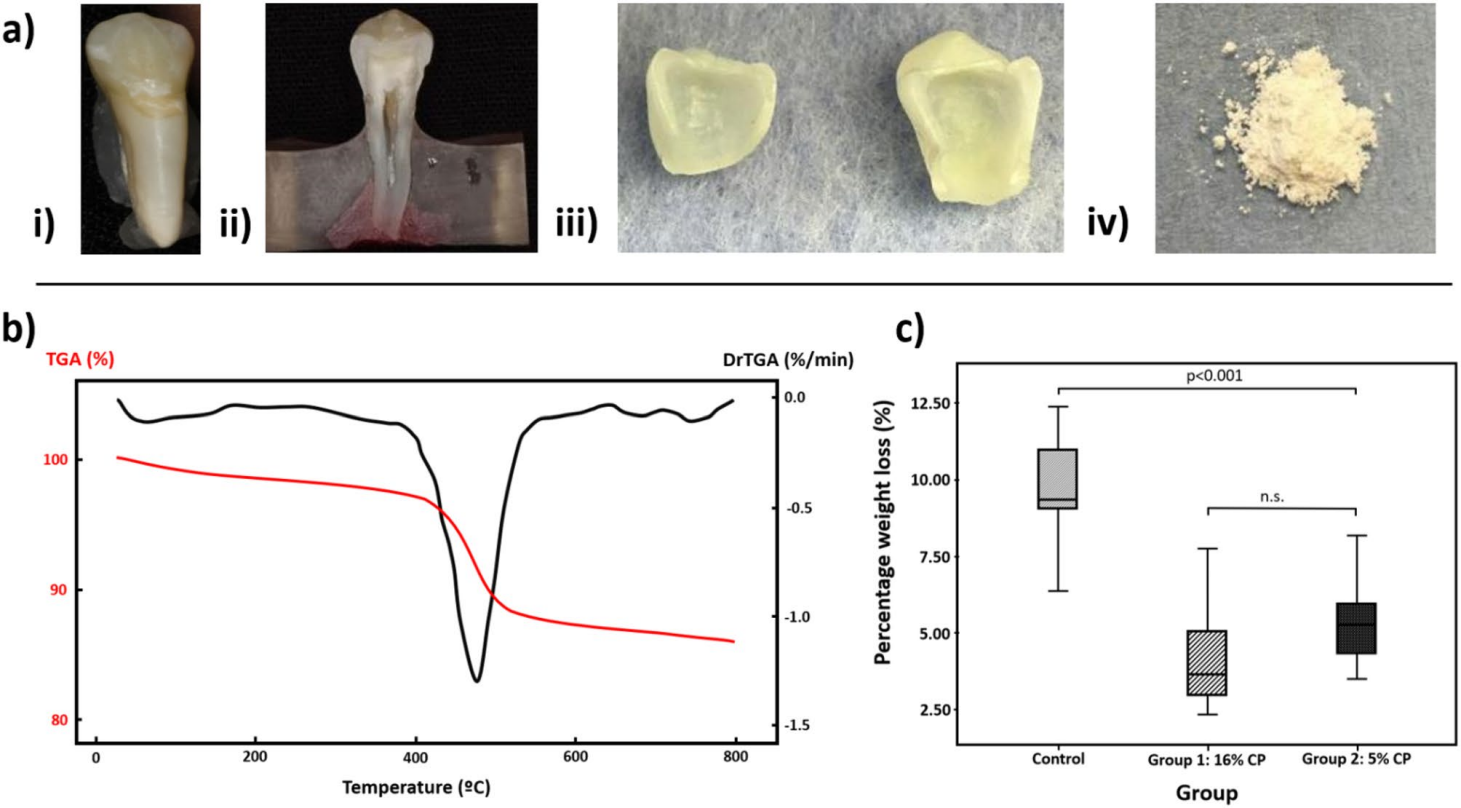
Enamel-protein (weight) ratio analysis pre and post-whitening. (**a**) Sample preparation for enamel-protein (weight) ratio Analysis. (i) Intact tooth sample. (ii) Longitudinally sectioned tooth into buccal/lingual half. (iii) 0.3–0.5 mm thick enamel shell. (iv) Enamel shell fine powder for thermogravimetry testing. (**b**) TGA curve (red) and its derivative (black) showing the mass change in enamel as a function of temperature ramp. **(c**) Box and Whisker plot, showing the range of TGA results in a percentage of weight loss for each group. A significantly lower (p < 0.001) enamel-protein content (by weight) of the treatment groups compared to the control.

Following extraction, the teeth were initially stored in a 70% ethanol solution for up to 5 days at room temperature before being debrided from remaining soft tissues and finally stored in a 0.1% thymol solution at 4 °C until required for the study (storage did not exceed 2 months) (Fig. 2a-i).

All methods were carried out in accordance with relevant local guidelines and regulations from both University College London (UK) and the University of Toronto (Canada).

### Sample preparation for enamel-protein (weight) ratio analysis

Thirty teeth were assigned randomly to control and two treatment groups (N = 10/group). This study's power was set at 80% with a p-value of 0.05 by estimating the required sufficient sample size in a pilot study. A high (16%) and the low (5%) concentration of CP was selected to follow dentist directed home teeth-whitening as recommended by the Council of European Dentists (CED-DOC-2012-061-E August 2012 Guidelines). The teeth were exposed to 5% or 16% CP gel for 4 h daily for 2 weeks and were kept in artificial saliva between treatments. The control group was kept in artificial saliva over the same period. The peroxide gel was homogeneously dispensed into an individual thermoplastic vacuum-formed (non-spaced) tray for each tooth and excess removed as recommended by the manufacturer. Although, teeth varied in size, we ensure that the crown of each tooth was completely immersed in the CP gel for the duration of the treatment. The artificial saliva was prepared using the ingredients outlined by McKnight-Hanes and Whitford^16^ (Table 1) and kept at 4 °C following preparation. Following treatment, each tooth was longitudinally sectioned into buccal and lingual halves (Fig. 2a-ii) that underwent complete dentin, pulp, and EDJ removal using diamond and stainless-steel burs in a fast turbine handpiece. The remaining enamel shell (0.3–0.5 mm thick) was cleaned in ultrasonic deionized water for 30 s (Fig. 2a-iii). The enamel shells were then pulverized to a fine powder for thermogravimetry testing using a “Spartan, Vibratory Sieve Shaker” (FRITSCH GMBH, GERMANY) (Fig. 2a-iv).

**Table 1:** Constituents of artificial saliva^16^.

Artificial saliva	
Constituents	g/L
Methyl-p-hydroxybenzoate (Na salt)	2.3
Sodium Caboxymethyle Cellulose	10
KCL	0.625
MgCl_2_·6H_2_O	0.059
CaCl_2_·2H_2_O	0.166
K_2_HPO_4_·3H_2_O	1.040
KH_2_PO_4_	0.326

Adjustment of pH to 6.75 using 0.1 M, HCL.

### Enamel-protein (weight) ratio analysis pre and post-whitening

Thermogravimetric analysis (TGA) was carried out using the TGA 50 analyzer (SHIMADZU CORPORATION, JAPAN). Each sample consisted of (4.00 ± 0.25) mg of powdered enamel placed in a platinum crucible. The TGA cycle was run between room temperature and 800 °C, at a rate of 10 °C/min with a 1-min hold at 30 °C. The measurements were carried out under oxygen (50 ml/min). The TGA curves are presented as a percentage of weight loss on the Y-axis (TGA%) and temperature (°C) in Fig. 2b.

### Cell culture

#### Impact of CP on human dental pulp stem cells (HDPSCs) survivability

**Figure 3:**
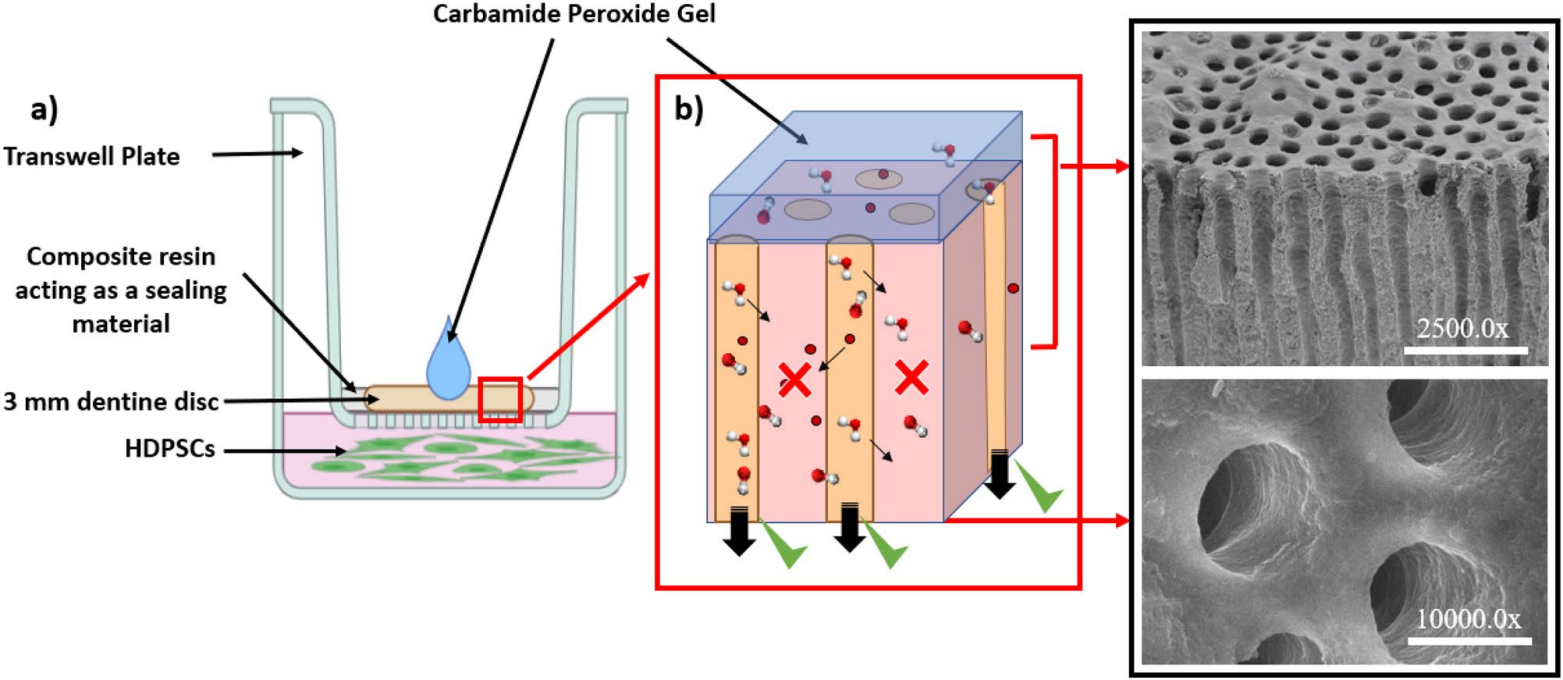
(**a**,**b**) Schematic depicting a cultured sample of HDPSCs exposed indirectly to CP drop through dentine disc.

**Figure 4:**
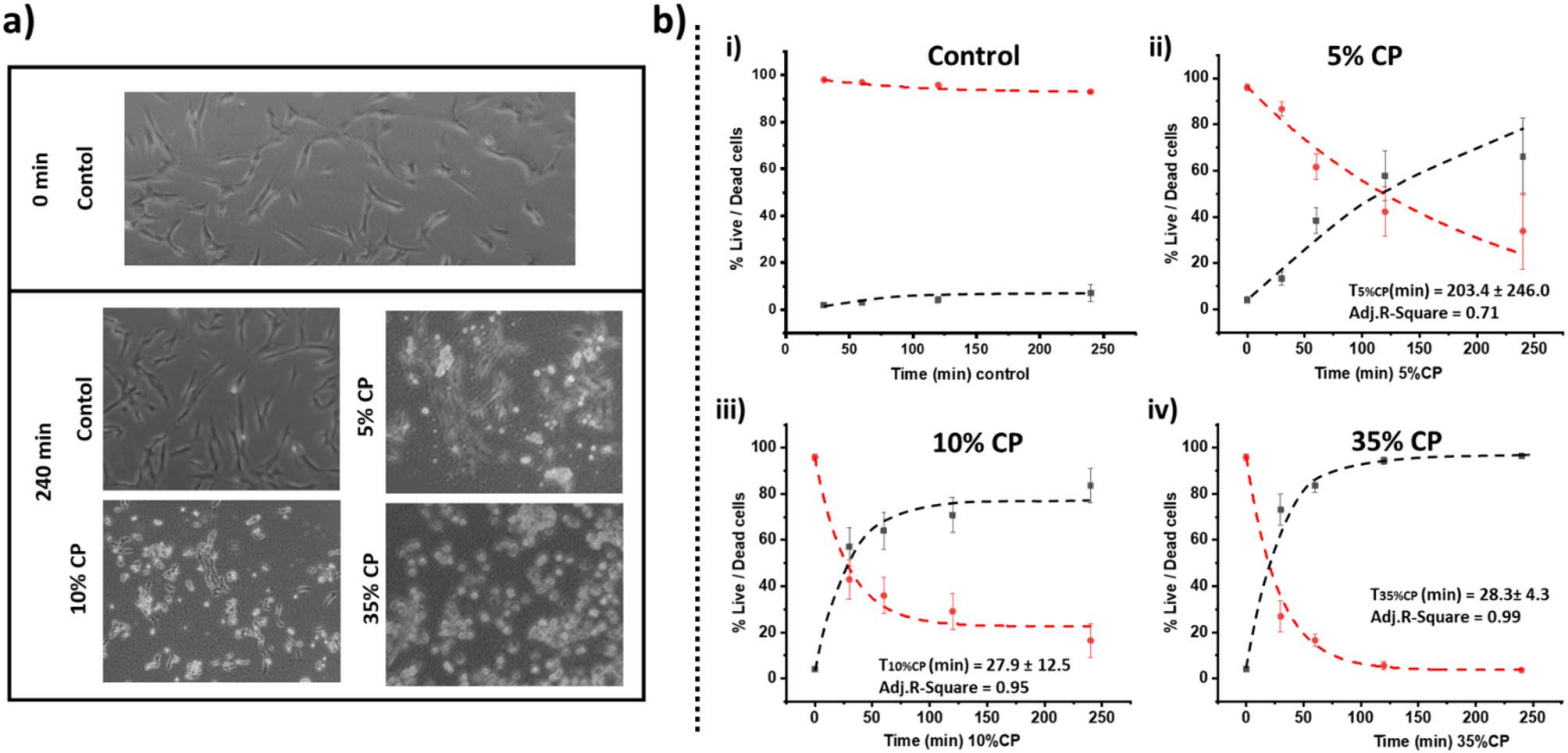
Impact of CP on human dental pulp stem cells (HDPSCs) viability. (**a**) Microscopic imaging showing the change in cell shape and morphology in the treatment groups compared to control. (**b**) Fitted plots showing the percentage of live/dead cells over time for the control group (no CP exposure) (i) and the three treatment groups (ii-iv) with exponential decay over the population of live cells (in red) and dead cells (in black).

Dentin discs were obtained from a total of 34 human teeth. The teeth were sectioned transversely at the mid-coronal level to obtain standardized dentin thickness of 3 mm-thick disc samples using a diamond microtome (STRUERS, ACCUTOM-50, STRUERS LTD., SOLIHULL, WEST MIDLANDS, UK). The discs were immersed in 37% phosphoric acid in a sonic bath for up to 15 s to remove the smear layer, followed by 2 min of rinsing in distilled water^17^. The dentin tubules' vertical orientation and their opening were checked by Scanning Electron Microscopy (FLEXSEM 1000. HITACHI HIGH TECHNOLOGIES; TORONTO, CANADA) on selected discs. Cell culture well-plates were modified with a transwell insert (THERMO FISHER SCIENTIFIC, WHITBY, CANADA) to support the 3 mm-thick dentin discs with a diameter < 4 mm (Fig. 3). Gaps between the transwell insert walls, and the edges of the dentin discs were sealed using a flowable composite resin material (FILTEK SUPREME ULTRA FLOWABLE RESTORATIVE, 3M ESPE) (Fig. 3a) to ensure that any CP gel deposited on top of the dentin disc could only perfuse through the dentin tubules (Fig. 3b) Additionally, we ensured that the cell growth medium was in direct contact with the dentin disc's underside to mimic the partial pressure in the dentinal tubules. HDPSCs (LONZA WALKERSVILLE, INC. MD 21793-0127 USA) were cultured in the dental pulp stem cell (DPSC) basal medium supplemented with dental pulp stem cell growth supplement (DPSCGS), 50 ml; l-glutamine, 10.0 ml; Ascorbic Acid, 5.0 ml; Gentamicin/Amphotericin-B (GA) (LONZA WALKERSVILLE, INC. MD 21793-0127 USA). Cells from the 4th passage were used with a minimum of 50,000 cells present in each well of the 12 well-plate the day before the treatment. The HDPSCs sub-cultures and dentin discs were randomly assigned to three treatment groups: exposure to 5%, 10%, 35% CP gel, and control. For the treatment groups, a drop of activated CP gel (using a drop of artificial saliva) at relevant concentrations was directly applied on top of the dentin disc mounted in the transwell insert (Fig. 3a) to simulate the exposure of the HPSCs to the whitening treatment. Artificial saliva was used for the control group. A time assay of HDPSCs survivability was performed for up to 4 h by evaluating the change in cell shape and morphology optically, as presented in Fig. 4a**.** Additionally, the ratio of live/dead cells at each time point was obtained by hemocytometry after trypan blue staining.

#### Impact of H_2_O_2_ on human gingival fibroblast cells (HGFCs) survivability

**Figure 5:**
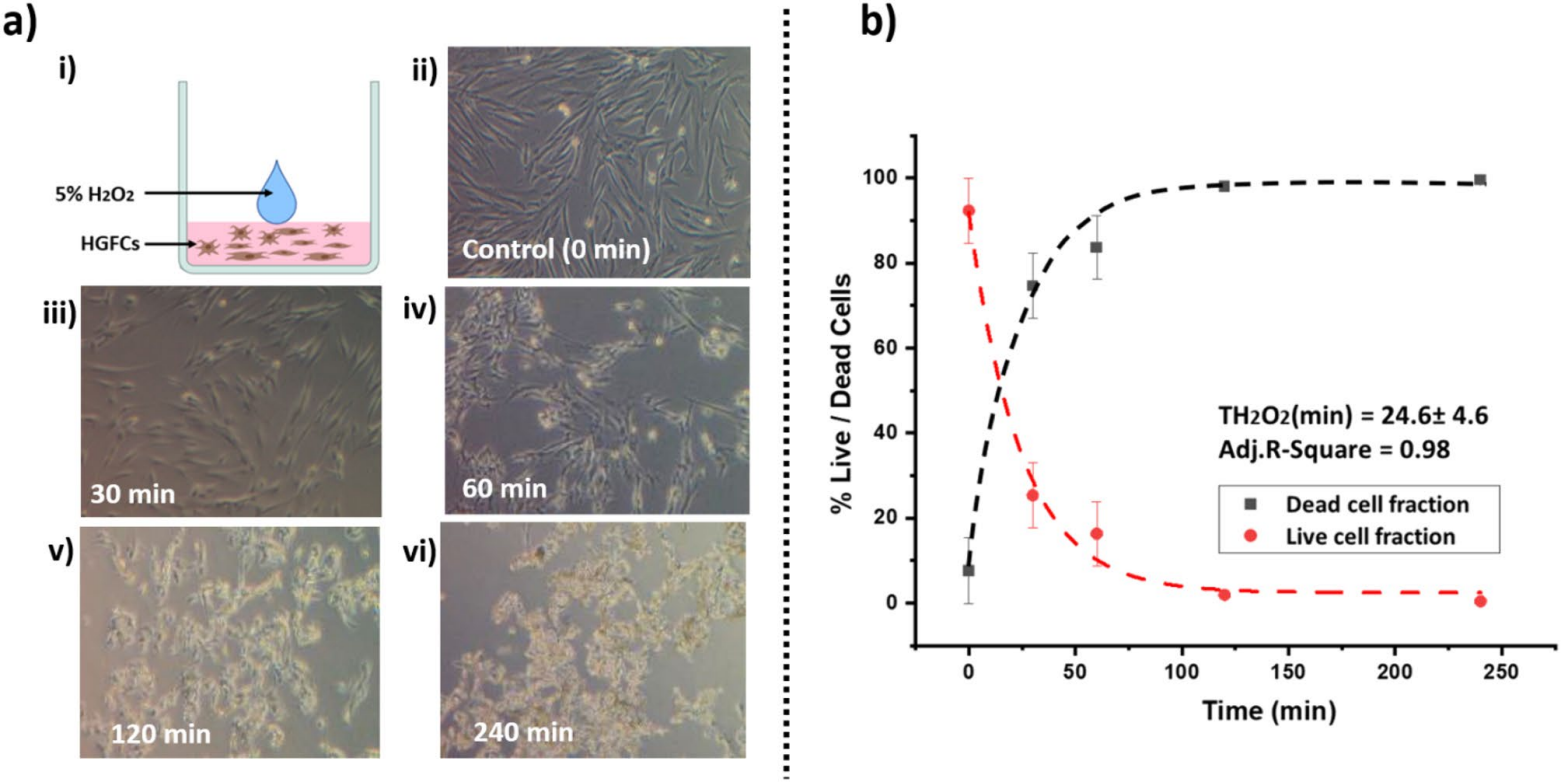
Impact of H_2_O_2_ on human gingival fibroblast cells (HGFCs) viability. (**a**) Evaluation of HGFCs exposed to 5% H_2_O_2_. (i) Schematic depicting a cultured sample of HGFCs exposed directly to 5% H_2_O_2_ solution. (ii, iii, iv, v & vi) Microscopic imaging showing the change in cell shape and morphology at different time points following exposure to H_2_O_2_. (**b**) Fitted plot showing the percentage of live/dead cells over time following the exposure of HGFCs to 5% H_2_O_2_.

HGFCs (SCINCELL RESEARCH LABORATORIES, CARLSBAD, CA 92008, UNITED STATES) were cultured in Dulbecco's Modified Eagle Medium (DMEM) (SIGMA CHEMICAL CO., ST. LOUIS, MO) supplemented with 10% fetal calf serum (GIBCO, GRAND ISLAND, NY) and 10% antibiotics at 37 °C (ISOTEMP FISHER SCIENTIFIC, PITTSBURGH, PA). Cells from the 4th passage were used with a minimum of 50,000 cells present in each well of the plate (12 wells) the day before treatment. Cultured HGFCs were exposed directly to 5% H_2_O_2_ solution, as depicted in Fig. 5a-i. A time assay of HGFCs survivability was performed for up to 4 h by evaluating the change in cell shape and morphology optically as presented in Fig. 5a-ii, iii, iv, v & vi**.** Additionally, the ratio of live/dead cells at each time point (30 min, 1 h, 2 h, and 4 h) was obtained by hemocytometry after trypan blue staining.

### Statistical analysis

For the enamel-protein (weight) ratio analysis, a pilot study (2 groups–4 repeats) was performed to assess the power. The power for the study was set at 80 with a p-value of 0.05, and the data were analyzed by a 2-sided test comparing 2 independent means with unequal variances and known standards of deviation using Stata 14 (STATACORP LP, Texas, USA). The clinical significance factor for this study was set as 10% change in mean. The estimated sample size was obtained using Satterthwaite Welch's t-test with the null hypothesis described as equal means. Upon completing data collection on the powered study, descriptive statistical analysis was first used to establish the mean and standard deviation for each group and present the data graphically as boxplots. The sample group means were then compared using a One-Way ANOVA with Bonferroni post-Hoc correction. The significance level for this comparison was set at p < 0.001. SPSS software (IBM SPSS STATISTICS 24, IBM CORP.) was used for these analyses.

For the cell culture: All cell cultures were conducted in experimental triplicate from stock. At each time point, the live and dead cell fraction was normalized to the percentage (%) of the total cell counted. The %Live cell was fitted with an exponential decay to calculate the 1/e time constant using Origin Pro 9.0 (ORIGINCORP, USA).

### Author disclaimer

The authors have purposely decided not to disclose the brand or manufacturer of the commercially available products used in this study was done in our previous submission^15^.

## Results

### Enamel permeability

Figure 2b shows a typical thermogravimetry (TGA) curve of weight change with temperature for the selected enamel sample. This change is shown more clearly in the DrTGA curve (1st derivative of the TGA curve), presenting the largest rate of mass change at T = 464 °C. The 16% CP treatment group showed an organic variation of 4.0%, whereas the 5% CP treatment group showed a variation of 5.4% (Fig. 2c) with no significant difference. Both treatment groups exhibited significantly lower (p < 0.001) enamel protein content by weight following CP treatment when compared to the control group (~ 50% on average).

### HDPSCs

After 240 min, HDPSCs, exposed all CP treatments, presented non-native cellular morphological appearances (round) when compared to the control (no exposure to CP). Cell death was confirmed by hemocytometry counting using trypan blue stain (Fig. 4a). Figure 4b presents the fitted plots of the percentage of live/dead cells over time for the control group (no CP exposure) (Fig. 4b-i) and the three CP gel concentrations (Fig. 4b-ii, iii & iv). There was an exponential decrease in the number of live cells remaining over time for the three treatment groups. By plotting the percentage of live/dead cells over time (Fig. 4b), it is possible to fit an exponential decay over the population of live cells remaining. Upon calculating the decay constant (1/e or of 70% drop in the original population) for each of the groups, it appears that both the T_10%CP_ and T_35%CP_ are close to one another (27.9 ± 12.5) min and (28.3.6 ± 4.3) min (Fig. 4b-iii & iv), respectively. In contrast, T_5%CP_ = (203.4 ± 246.0) min (Fig. 4b-ii).

### HGFCs

HGFCs start to lose their morphological appearances after 30 min and cell death was confirmed by hemocytometry counting using trypan blue stain. Over 95% cell death can be recorded after 120 min exposure to H_2_O_2_. The 1/e time constant occurs at T_H2O2_ = (24.6 ± 4.6) min (Fig. 5b).

## Discussion

### Enamel permeability

Enamel encounters whitening agents firstly. However, enamel consists of > 98% of a hydroxyapatite mineral phase, and < 2% of the enamel consists of protein (90% amelogenin, 10% enamelin, and ameloblastin). These proteins form an enamel sheath around the enamel rods in mature teeth^18^. Any loss in these proteins would increase the enamel permeability and promote extrinsic reagents' penetration^19^. Our results suggest that enamel protein is susceptible to oxidative degradation from the CP breakdown reaction by-products. The main mass change (430–500 °C) reflects the oxidative degradation of the enamel's organic content leaving only enamel mineral content in the crucible^20^. A low CP (5%) concentration is sufficient to alter the protein content in the enamel. Peroxide-based whitening agents have been shown to induce alterations of the surface texture and morphology of enamel^8,21^. Ferreira et al. demonstrated that 35% hydrogen peroxide (HP) affected enamel morphology, producing porosities, depressions, and superficial irregularities to various degrees^22^. CP specifically induced uniform etching-like erosion of surface and subsurface enamel due to mineral dissolution and decalcification^23^. It can also cause a decrease in enamel hardness and higher enamel roughness^24^. An electron microscopy study observed that whitening treatment with either CP or HP induced various surface alterations, including reducing the prismatic layer, demineralization of the enamel prisms, and greater porosity within and between the enamel prisms^25^. Our approach extended this finding as we confirmed that enamel's protein content is also significantly reduced, asserting the opening of the inter-prismatic spaces that would act as conduits for the penetration of CP breakdown reactions by-products to the inner part of the tooth, including the pulp. However, as changes in enamel composition can be reversible in-vivo, this alteration in the enamel composition is not final.

### The effect of teeth-whitening on HDPSCs and HGFCs

Dental hypersensitivity occurs in about two-thirds of patients during vital bleaching^26^. It is principally attributable to peroxide diffusion into the enamel and dentin, resulting in dehydration and subsequent fluid movement in the dentinal tubules, which stimulates the nerve endings, leading to sensitivity^14^. The over-exposure of the by-products released from the whitening gel to most cells causes oxidative stress^27^. An increase in ROS (Reactive Oxygen Species) levels causes deleterious effects on several cell components, including lipid peroxidation, oxidative alterations of protein, and DNA cell damage^28^. Decay constants can be used to indicate when the culture is no longer viable. Interestingly, both 10% and 35% CP exposure impacted the cell culture similarly, whereas 5% CP exposure affects the culture to a much lesser extent, as proven by the wider error on the constant value. Translating these findings in-vivo may results in the partial mitigation of this rapid cell death due to positive pulp pressure, dental fluid, and the cell's inherent defence to oxidative stress. One of the limitations of our study was not to investigate cellular recovery and host immune response following exposure to H_2_O_2_-derived free radicals.

#### HDPSCs

It is known that HDPSCs represent a heterogeneous culture from pulp tissue, including a population of mesenchymal stem cells^29,30^. We decided to use this heterogeneous culture for this investigation as mesenchymal stem cells are recruited as precursors to new odontoblast-like cells, responsible for dentin-pulp complex regeneration following lethal odontoblast damage as expected following exposure to ROS^31^. Odontoblasts are also involved in the initiation, development, and maintenance of the pulp inflammatory/immune response, representing the host's first defence line^32^. It is thus essential to evaluate the survivability of HDPSCs in a highly damaging environment. Our results suggest that both the 10% and 35% CP gel exposure to the dentin disc affect HDPSCs in the same manner (based on the time constant) as if the cells were exposed directly to the 5% H_2_O_2_ as done for HGFCs. The dentin disc (3 mm thick) cannot withhold the penetration of the CP breakdown reactions by-products to reach the cell culture when the CP's concentration exceeds 5% CP, as demonstrated by cell viability measurements. This is consistent with our previous in-vitro study^15^, which had proven that even with a low concentration (5% CP) whitening agent, the peroxide and free radicals could diffuse through the dentin into pulp tissue, causing collagen degradation and reduction in organic dentinal components (Amide I and Amide III). All teeth-whitening protocols evaluated in the present study resulted in trans-enamel and trans-dentinal peroxide diffusion, directly related to the teeth-whitening gel's concentration and the time of application to dentin. However, the pulp cells from human tissue were still highly sensitive to all bleaching protocols tested in this investigation, albeit the response to 5% differed from 10% and 35%. Previous studies demonstrated pulp cell oxidative stress induced by H_2_O_2_ in a time-/concentration-dependent manner^10,33^. In vital teeth, oxidative stress causes inflammatory pulp response directly related to the enamel's and dentin’s thickness of bleached teeth^31,34^. A study by Sato et al. demonstrated that in-vivo oxidative stress generated by a 35%-H_2_O_2_ gel in the pulp tissue of young human premolars increased the activity of metalloproteinases and cysteine cathepsin B, which both play an essential role in protein matrix degradation. According to data from the present study, these negative side-effects may be minimized by shortening the contact time with the enamel or dentin or reducing the concentration of H_2_O_2_ in teeth-whitening agents. Several clinical trials have shown that teeth-whitening gels with H_2_O_2_ concentration (15–20%) applied to enamel for 45–60 min can promote a significant color change caused by a high 35% H_2_O_2_ gel concentration. In these studies, tooth sensitivity incidence ranged from 24 to 78%, considered mild in severity^35–37^. Moreover, a more recent clinical study revealed that the whitening efficiency of low 5% CP gels were as effective as those containing 10% CP^38^. Thus, by reducing the concentration of peroxide and exposure time, it is possible to execute an effective and less aggressive home tooth-whitening protocol. However, in-vivo studies in vital human teeth are needed to assess tooth-whitening effectiveness and pulp responses after applying the teeth-whitening protocols evaluated in the present study. Although our study did not intend to replicate an ‘in-vivo” like tooth model, we produced a simple perfusion model that facilitates testing the impact of whitening approaches on dental cells to evaluate their short-term viability.

#### HGFCs

Human gingival fibroblasts play an essential role in the structure of tissues, function and immune defence of the host^39^. Some reports indicate that H_2_O_2_ caused irritation, ulceration, burning and certain adverse effects on the gums^40,41^. It was reported that hydrogen peroxide promoted PKC and ERK 1/2 activation and decreased cell viability^42^. An in-vitro study reported that hydrogen used at concentrations of 10 to 200 mM promoted apoptosis. The characteristic apoptotic events such as morphological changes, including chromatin condensation, nuclear and DNA fragmentation, considered a hallmark of cells undergoing apoptosis, were detected in hydrogen peroxide HGFCs^43^. In the present study, we found that 5% H_2_O_2_ reduced the viability of HGFCs. The immediate impact of H_2_O_2_ in gingival fibroblast viability does not directly reproduce events in the pulp chamber but indicates why gingival irritation can occur and supports limiting direct contact with the gingiva^44^. Reversal of this gingival effect has been reported after 2 weeks with 10 and 16% CP^45^.

## Conclusions

Within the limitations of this in-vitro model, we can conclude that 5% and 16% of CP induces a 50% reduction in the percentage weight of organic content in enamel, making it more porous and susceptible to peroxide diffusion into dentin and pulp tissue. Moreover, home tooth-whitening with high concentration and long application time (35% CP gel applied for 4 h) can produce intense oxidative stress on both gingival fibroblast and pulp cells, associated with a severe reduction in cell viability. From our data, using lower concentrations of CP (5%) would be less harmful for the dental cells and therefore should be the recommended concentration. Yet, the use of increased CP concentrations is sometimes preferred by the end-users to see an immediate whitening effect on the teeth. Our study demonstrated an extreme side-effect of CP exposure to dental cells, leading to rapid cell death when using such high concentrations of CP. As a result, a compromise needs to be found between the concentration of CP used, exposure time, desired patients’ outcomes, and, finally, side-effects experienced. This compromise should be tested in-vivo prior to market release and patients should be made aware of the impact of such procedure on their oral health.
